# Access, socioeconomic environment, and death from COVID-19 in Nebraska

**DOI:** 10.3389/fpubh.2022.1001639

**Published:** 2022-10-06

**Authors:** He Bai, Michelle Schwedhelm, John-Martin Lowe, Rachel E. Lookadoo, Daniel R. Anderson, Abigail E. Lowe, James V. Lawler, M. Jana Broadhurst, David M. Brett-Major

**Affiliations:** ^1^Department of Epidemiology, University of Nebraska Medical Center, Omaha, NE, United States; ^2^Global Center for Health Security, University of Nebraska Medical Center, Omaha, NE, United States; ^3^Nebraska Biocontainment Unit, Nebraska Medicine, Omaha, NE, United States; ^4^Department of Environmental, Agricultural and Occupational Health, University of Nebraska Medical Center, Omaha, NE, United States; ^5^Center for Preparedness Education, University of Nebraska Medical Center, Omaha, NE, United States; ^6^Division of Cardiovascular Medicine, Department of Internal Medicine, University of Nebraska Medical Center, Omaha, NE, United States; ^7^Center for Biosecurity, Biopreparedness, and Emerging Infectious Diseases, University of Nebraska Medical Center, Omaha, NE, United States; ^8^Division of Infectious Diseases, Department of Internal Medicine, Nebraska Medicine, Omaha, NE, United States; ^9^Department of Pathology and Microbiology, University of Nebraska Medical Center, Omaha, NE, United States

**Keywords:** healthcare access, length of stay, definitive care, mortality, COVID-19

## Abstract

Our study assesses whether factors related to healthcare access in the first year of the pandemic affect mortality and length of stay (LOS). Our cohort study examined hospitalized patients at Nebraska Medicine between April and October 2020 who were tested for SARS-CoV-2 and had a charted sepsis related diagnostic code. Multivariate logistic was used to analyze the odds of mortality and linear regression was used to calculate the parameter estimates of LOS associated with COVID-19 status, age, gender, race/ethnicity, median household income, admission month, and residential distance from definitive care. Among 475 admissions, the odds of mortality is greater among those with older age (OR: 1.04, 95% CI: 1.02–1.07) and residence in an area with low median household income (OR: 2.11, 95% CI: 0.52–8.57), however, the relationship between mortality and wealth was not statistically significant. Those with non-COVID-19 sepsis had longer LOS (Parameter Estimate: −5.11, adjusted 95% CI: −7.92 to −2.30). Distance from definitive care had trends toward worse outcomes (Parameter Estimate: 0.164, adjusted 95% CI: −1.39 to 1.97). Physical and social aspects of access to care are linked to poorer COVID-19 outcomes. Non-COVID-19 healthcare outcomes may be negatively impacted in the pandemic. Strategies to advance patient-centered outcomes in vulnerable populations should account for varied aspects (socioeconomic, residential setting, rural populations, racial, and ethnic factors). Indirect impacts of the pandemic on non-COVID-19 health outcomes require further study.

## Introduction

Severe acute respiratory syndrome coronavirus-2 (SARS-CoV-2), and its coronavirus disease 2019 (COVID-19), has claimed the lives of 5.8 million people since its discovery with over 410 million cases recognized worldwide (1). The United States has surpassed all other countries in the emergency by hosting approximately a fourth of the total worldwide cases, ~77 million, with over 900 thousand deaths (2). As cumulative COVID-19 cases and deaths climb, life expectancy drops. When the death toll reaches 1 million, the US will have regressed to mortality conditions present 25 years ago (3).

The national conversation around COVID-19 and social vulnerability is reflected in the experience of Nebraska's rural and frontier counties exemplified by critical access care settings, migrant and immigrant critical infrastructure workforces, as well as metropolitan diaspora. Our research efforts with the first recognized COVID-19 cohort in the U.S. examined potential associations between vulnerabilities and outcomes in participants (4). This led to a broader examination of access to care related to patient-centered outcomes. Current research examining the COVID-19 pandemic shows that socioeconomic burdens, such as income inequality, correlated with greater number of deaths among the financially vulnerable (5). Increased mortality was also seen among minority populations, attributing to higher risks of comorbid diseases that increases the risk of poor outcomes among patients (6). However, there is still little evidence regarding how specific determinants of access to care (e.g., physical distance and social aspects) may impact COVID-19 related outcomes. We pursued this issue.

## Methods

Hypotheses were generated through exploration of data from University of Nebraska Medical Center's (UNMC) Clinical Characterization Protocol for Severe Emerging Infectious (CCPSEI) cohort study, single IRB no., 146-20-FB (7). Then, de-identified data were obtained on inpatients admitted with suspected or confirmed COVID-19 at Nebraska Medicine, Omaha, Nebraska, between April and October 2020, inclusively. Inpatients who had negative COVID-19 test results were included as the control group for analysis and restricted to those who had a diagnostic code for sepsis or non-hospital acquired pneumonia. Confirmed COVID-19 inpatients had a positive SARS-CoV-2 nucleic acid test on either conventional real-time PCR, Roche Cobas, or BioFire FilmArray^®^. Persons with COVID-19 were included only on their first presentation of COVID-19. Persons without COVID-19 could be included more than once due to opportunities for re-admission if admission were in different months. Arrival to Nebraska Medicine's main campus in Omaha was considered definitive care. The data set included disposition at discharge, length of stay, gender, age, admission month, race, and COVID-19 test status, as well as transformed zip code, median household income in residential area, and distance from definitive care as follows.

Zip codes were used for the purpose of determining distance from definitive care with a focus on the challenges of critical access settings (rural, sometimes frontier areas). However, because individual patient zip codes were determined to breach patient confidentiality, zip codes were grouped as belonging to Omaha or Lincoln metropolitan areas, or, if outside of these metropolitan areas, by Nebraska state senate districts as determined by the Nebraska legislature website and given to the UNMC health record access coordinator for data extraction. A senate district was split into two groups if it included more than 5 zip codes. Districts with <3 zip codes were clustered together by geographical proximity. These groupings then were placed in bands of 50, 100, 200, 300, or 400 miles from definitive care, determined using the nearest edge of the district or clustered areas in Google^®^ Maps. There were no observations in the 400 miles band, excluding it from further analysis. Zip codes that were not from Nebraska were categorized as “Not in Nebraska.” These also were not analyzed and included neighboring states as well as occasional regional and national referrals from other areas.

For the approximate 78 zip codes associated with Omaha and Lincoln *via* the United States Postal Service zip code lookup tool, income categorizations were included as the state senate districts were sufficiently identified with appropriate median household income from Census data (8, 9). If districts were clustered together because they had <3 zip codes, the average of the median household income of the districts was used.

### Statistical analysis

All analysis was performed on SAS^®^ OnDemand for Academics. The outcome variables were mortality and length of stay. Covariates were subjected to backwards elimination to identify the statistically significant associations, then confirmed using bivariate analysis. The final set of covariates were depicted through multivariate analysis using logistic regression. The association between mortality and the covariates were measured using odds ratio. The adjusted multivariate analysis was fitted using linear regression due to the continuous nature of length of stay and the parameter estimates. The association between length of stay and the covariates are measured using parameter estimates. A *p*-value of <0.05 was considered to be statistically significant accounting for the degrees of freedom in the collapsed transformations. Figures were created on Microsoft Excel^®^ 2016 and SAS^®^ OnDemand for Academics.

### Patient and public involvement

No patients or the public were involved in the study due to our primary data incorporating only deidentified data.

### Ethics approval

The protocol for the CCPSEI study involving human participants was approved by the Institutional Review Board of the University of Nebraska Medical Center (single IRB no., 146-20-FB) and was conducted in accordance with the Declaration of Helsinki.

## Results

**Figure 1:**
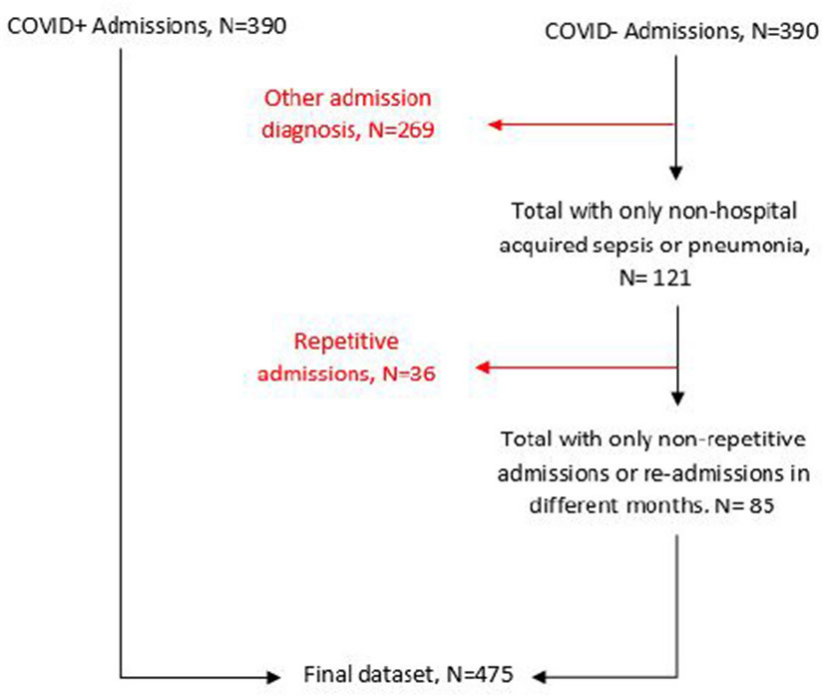
Flow chart for dataset cleaning and analysis.

Out of 780 identified patients, 85 admissions of suspected but ultimately not confirmed COVID-19 cases and 390 confirmed COVID-19 cases met selection criteria (Figure 1).

Among patients without COVID-19 (SARS-CoV-2 negative), there was an equal split of genders and an overall mortality of 9% (Table 1). COVID-19 negative patients had a median length of stay of 10 days and a mean of 15 days compared to a median length of stay of 6 days and a mean of 10 days among COVID-19 positive patients.

**Table 1:** Demographic characteristics by COVID-19 status among patients at Nebraska Medicine between April and October 2020, Omaha, Nebraska.

		**COVID-19 negative**			**COVID-19 positive**		
**Demographic characteristics**		***N*** = **85 (%)**			***N*** = **390 (%)**		
**Length of stay**		**IQR: 5–18**			**IQR: 4–12**		
		**Median: 10**			**Median: 6**		
		**Mean: 15.2**			**Mean: 10.1**		
		**All**	**Survived**	**Expired**	**All**	**Survived**	**Expired**
Gender	Male	43	39 (91%)	4 (9%)	210	186 (89%)	24 (11%)
	Female	42	38 (90%)	4 (10%)	180	165 (92%)	15 (8%)
Age	<45 years	17	15 (88%)	2 (12%)	94	93 (99%)	1 (1%)
	46–60 years	20	17 (85%)	3 (15%)	101	93 (92%)	8 (8%)
	61–70 years	24	22 (92%)	2 (8%)	103	93 (90%)	10 (10%)
	>70 years	24	23 (96%)	1 (4%)	92	72 (78%)	20 (22%)
Race	White	68	61 (90%)	7 (10%)	194	172 (89%)	22 (11%)
	Black	8	7 (87%)	1 (13%)	55	50 (91%)	5 (9%)
	Other	5	5 (100%)	0 (0%)	27	23 (85%)	4 (15%)
	Unknown	4	4 (100%)	0 (0%)	114	106 (93%)	8 (7%)
Distance from definitive care	Not in nebraska	22	20 (91%)	2 (9%)	46	41 (89%)	5 (11%)
	Within 50 miles	50	45 (90%)	5 (10%)	315	284 (90%)	31 (10%)
	Within 100 miles	7	6 (86%)	1 (14%)	16	15 (94%)	1 (6%)
	Within 200 miles	3	3 (100%)	0 (0%)	11	9 (82%)	2 (18%)
	Within 300 miles	3	3 (100%)	0 (0%)	2	2 (100%)	0 (0%)
Median household income in area of residence	Unknown	49	45 (92%)	4 (8%)	117	108 (92%)	9 (8%)
	<$50,000	24	20 (83%)	4 (17%)	196	173 (88%)	23 (12%)
	$50,000–$75,000	8	8 (100%)	0 (0%)	56	52 (93%)	4 (7%)
	>$75,000	4	4 (100%)	0 (0%)	21	18 (86%)	3 (14%)

**Figure 2:**
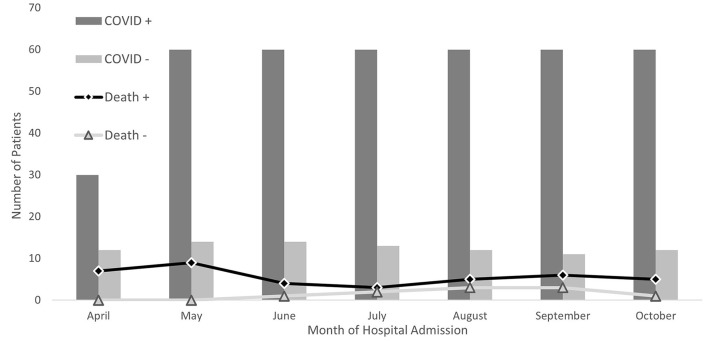
Hospital admissions and death by SARS-CoV-2 status among patients at Nebraska Medicine Between April and October 2020, Omaha, Nebraska.

Among COVID-19 patients there were 39 deaths (10%), 20 of which were among those aged >70 years old. The majority of patients were white and lived within 50 miles of definitive care. COVID-19 patients self-identified as Black race (14%) and other minorities (7%) at a greater rate than those patients not confirmed to have COVID-19 (9 and 6%, respectively). Patients without confirmed COVID-19 from areas with median household incomes <$75,000 died more frequently. There was a greater proportion of COVID-19 patients that were from an area with <$50,000 median household income (50 vs. 28% among those not confirmed with COVID-19). Most deaths among those without confirmed COVID-19 occurred in August and September (63%), while May had the greatest percentage of deaths among admissions who had COVID-19 (23%) (Figure 2).

Age was the only variable selected through backward selection; positive COVID-19 status, gender, race, distance from definitive care, and median household income were reintroduced to the model as literature relevant risk factors related to mortality (Table 2). With each year increase in age, the odds of mortality increased by a factor of 1.04. Being from a low wealth area carried 2.11 odds of increase in mortality compared to those whose residence have a median household income >$75,000. There was a consistent trend across distance bands for better outcomes with closer proximity to the medical center, although this did not achieve statistical significance besides those who reside within 200 miles, who have a 9.01 greater odds of mortality compared to those who live within 50 miles from definitive care. A Cochran Mantel Haenszel analysis testing a trend between mortality and month of admission did not achieve statistical significance (*p* = 0.85)

**Table 2:** Multivariate logistic regression factors associated with mortality among patients at Nebraska Medicine between April and October 2020, Omaha, Nebraska.

**Factors associated with mortality**		**Odds ratio**	**95% CI**	***P*-value**
COVID-19	Negative	–	–	–
	Positive	1.029	(0.437, 2.422)	0.948
Age		1.043	(1.020, 1.066)	<0.001
Gender	Male	–	–	–
	Female	0.702	(0.370, 1.333)	0.280
Race	White	–	–	–
	Black	0.671	(0.245, 1.838)	0.437
	Other	1.240	(0.371, 4.148)	0.727
	Unknown	0.618	(0.243, 1.573)	0.313
Median household income at residence	>$75,000	–	–	–
	$50,000–$75,000	0.854	(0.160, 4.547)	0.853
	<$50,000	2.105	(0.517, 8.569)	0.299
	Unknown	0.446	(0.087, 2.282)	0.332
Distance from definitive care	Within 50 miles	–	–	–
	Within 100 miles	1.839	(0.310, 10.903)	0.502
	Within 200 miles	9.011	(1.237, 65.665)	0.030
	Within 300 miles	<0.001	(<0.001, >999.999)	0.990
	Not in nebraska	2.943	(0.727, 11.919)	0.130

In multivariate analysis, length of stay statistically significantly increased by ~5 days if a patient was not confirmed to have COVID-19 (Table 3).

**Table 3:** Linear regression analysis for length of stay among patients at Nebraska Medicine between April and October 2020, Omaha, Nebraska.

		**Unadjusted**			**Adjusted**		
		**Parameter**	**95% CI**	***P*-value**	**Parameter**	**95% CI**	***P*-value**
		**estimate**			**estimate**		
COVID-19	0 = Negative	−5.125	(−7.874, −2.375)	<0.001	−5.109	(−7.920, −2.299)	<0.001
	1 = Positive						
Age		0.042	(−0.017, 0.101)	0.166	0.040	(−0.019, 0.099)	0.185
Gender	0 = Male	−1.911	(−4.046, 0.224)	0.079	−1.990	(−4.112, 0.131)	0.066
	1 = Female						
Month	1 = April	−0.215	(−0.775, 0.345)	0.451	−0.121	(−0.680, 0.438)	0.671
	2 = May						
	3 = June						
	4 = July						
	5 = August						
	6 = September						
	7 = October						
Race	0 = Unknown	0.369	(−0.956, 1.694)	0.585	0.131	(−1.194, 1.455)	0.846
	1 = White						
	2 = Black						
	3 = Other						
Distance from definitive care	0 = Not in nebraska	0.198	(−1.483, 1.879)	0.817	0.292	(−1.388, 1.972)	0.733
	1 = Within 50 miles						
	2 = Within 100 miles						
	3 = Within 200 miles						
	4 = Within 300 miles						
Median household income at residence	0 = Unknown	−0.110	(−1.404, 1.183)	0.867	0.164	(−1.136, 1.464)	0.804
	1 = <$50,000						
	2 = $50,000–$75,000						
	3 = >$75,000						
							

**Adjusted full model:** Length of Stay = 13.73 – 5.11 (COVID-19) + 0.04 (Age) - −1.99 (Gender) - −0.12 (Month) + 0.13 (Race) + 0.29 (Distance from Definitive Care) + 0.16 (Mean Household Income). **Adjusted reduced model:** Length of Stay = 16.21 – 5.19 (COVID-19).

Length of stay increased by approximately a day for each 25-year increase in age in the adjusted model and increased by 2 days if the patient was male. An increase in distance from definitive care is associated with an increase in length of stay for both the adjusted and unadjusted model, however, while low median household income is associated with greater LOS in the unadjusted model, the relationship is reversed when other risk factors are considered.

## Discussion

We observed both increased COVID-19 burden and higher mortality with age as well as with those who resided in areas with median household income <$50,000. This is consistent with previous reports that suggest marginalized communities experience a greater number of deaths from COVID-19 (5, 10–15). Age is widely recognized strong risk factor for COVID-19 mortality. The impact of wealth, however, requires additional research in order to dissect the related factors that are exacerbated by low socioeconomic status. Some analyses of wealth have showed similar results, and concurrent non-communicable disease and reduced healthcare access are relevant risks for poor outcomes in SARS-CoV-2 (16–18). The Omaha metropolitan area as well as outlying and other rural communities in Nebraska are home to many crucial service and essential industry workers, such as those working within meat processing facilities, labor sectors in which minorities are over-represented. Poor and crowded living conditions, underlying health conditions including those complications linked to late identification and decreased healthcare access, and unfavorable working conditions play a role in increased confirmed COVID-19 cases and mortality (19). Understated impacts from housing instability, reduced food security, and the psychological tolls of the pandemic require further study, and could be impacting elements of wealth and distance effects in our study (20, 21).

Surprisingly, those with COVID-19 had shorter lengths of stay. We expected length of stay to be longer in these individuals due to delays in disposition related to actual and perceived risks of communicability, though pressures from hospital stress and need for available isolation care space may have modulated this. This finding also may relate to delays related to initial requirements for assessment in isolation care, with consequential delays in access to services for non-COVID-19 conditions. Our observation of longer length of stay for men may be partially explained by others' observation of greater COVID-19 severity (22). Age was not a significant factor in length of stay. A relative increase in presentation of younger patients due to the pandemic may have contributed to this observation.

We observed that among more rural communities, the farther the residence from definitive care, the greater the mortality and the longer the length of stay for a patient. These trends did not reach statistical significance, but the potential effects of delay in care and difficulty in disposition remain important avenues for exploration in our setting where frontier counties still exist. Follow up work is indicated incorporating socio-demographic factor data subject to validation so that such patterns can be assessed across subsequent years of the pandemic in a broad range of communities.

*Taken together with the impacts of wealth and the minority trends that we observed, access perturbed for any reason matters in patient-centered outcomes in the pandemic*.

### Limitations

Our research was subject to limitations. Full characterization of demographic risks for COVID-19 was not in scope for the cases in this study. There were 118 individuals within the expanded dataset that had unknown race and ethnicity, and persons self-identified as Hispanic (an important critical infrastructure constituency in our region) were not well-identified. Those with Hispanic ethnicity were represented to or above population levels among admissions for suspected and confirmed COVID-19 disease (separate electronic health record quality review, data not shown), and so were represented in our data set. Though access to care and its impact on LOS and mortality is recognized, our study would benefit from a greater population of patients that travel >50 miles for definitive care to further assess distance as a risk factor for both COVID-19 and non-COVID-19 outcomes. Income was established by analyzing the median household income for each state senate district through an average rather than precise household determinations, and only in the Omaha and Lincoln metropolitan areas. Because our dataset lacked sufficient information to look at distance in urban travel, distance from the medical center should not be construed as applying to differences between affluent and poor communities within the metropolitan areas, including issues around urban transit. These challenges may have attenuated observed financial disparities and confounded the impact of distance (being from a remote rural area). The potential impact of having health insurance, its type and quality, is not known in this data set. The expanded dataset included all hospitalized patients without discriminating disease severity, and later outcomes from COVID-19 patients with serial admissions were not captured. Uniformly little societal measures were in place in Nebraska during the censuring period, and an assessment of comparative impact of their presence or absence was not possible.

## Conclusion and public health implications

The impact that health inequity has on patients is clearly evidenced by longer lengths of hospital stay and greater odds of mortality. As previously stated, crowded living conditions and unfavorable, often times dangerous, working conditions among those with lower socioeconomic status brings forth COVID-19 related health risks that are further exacerbated by other factors related to health disparities. Low socioeconomic status individuals, particularly minorities, face other barriers including food insecurity, lack of information, isolation, increased and lack of healthcare access that also impact patient centered outcomes (23). Minorities also face other determinants of health that can present barriers for care such as lack of insurance, limited language English proficiency, racial discrimination, prevalence of other comorbidities, and filial obligations to family members outside of the nuclear home that increases financial strain (24). Age is also a known risk factor related to mortality, however, the elderly population still face similar barriers to healthcare access that other marginalized communities have.

The United States ranks first in the global health index, and yet our postulated readiness for a health emergency has met SARS-CoV-2 with failure in 2020. There are myriad reasons for this, but one highlighted in this study must be attacked. ***Access to care in every context*** ***must be addressed*. **Directing measures to target the care access gap related to the elderly, those with less wealth, and potential social and physical barriers including distance and transport, must be a main focus of preparedness and response activities in order to decrease mortality as well as the system costs of length of stay. Other research exploring the consequences of being in isolation care must also weigh on these disparities when more time in isolation has resulted. In addition to the focused application of resources such as financial support for dwelling and food security, safe space and pre-hospital access for features like testing, targeted and innovative risk communication, and social mobilization must be applied as part of the tool set to overcome barriers in access (12, 23, 25–27). Our analyses also suggest that the pandemic may have a negative effect on non-COVID-19 outcomes. A better understanding of hospital and home stress, consequences of isolation care even in sophisticated healthcare settings and their numerous impacts on patient-centered outcomes is needed (28, 29).

## Data availability statement

The datasets presented in this article are not readily available due to privacy constraints. Requests to access the datasets should be directed to eteg@unmc.edu.

## Ethics statement

The studies involving human participants were reviewed and approved by UNMC IRB. The patients/participants provided their written informed consent to participate in this study.

## Author contributions

HB and DB-M conceptualized and designed the study. HB conducted the analysis and supervised by DB-M. DB-M and MB conceptualized the CCPSEI study which the article was based on. All authors assisted with interpretation of the results and contributed to the editing of the article.

## Funding

This study received intramural funding support.

## Conflict of interest

The authors declare that the research was conducted in the absence of any commercial or financial relationships that could be construed as a potential conflict of interest.

## Publisher's note

All claims expressed in this article are solely those of the authors and do not necessarily represent those of their affiliated organizations, or those of the publisher, the editors and the reviewers. Any product that may be evaluated in this article, or claim that may be made by its manufacturer, is not guaranteed or endorsed by the publisher.
